# Preliminary Exploration of Swine Veterinarian Perspectives of On-Farm Euthanasia

**DOI:** 10.3390/ani10101919

**Published:** 2020-10-19

**Authors:** Lily N. Edwards-Callaway, Mary Caitlin Cramer, I. Noa Roman-Muniz, Lorann Stallones, Sofia Thompson, Sari Ennis, Jordan Marsh, Hailey Simpson, Elizabeth Kim, Elaine Calaba, Monique Pairis-Garcia

**Affiliations:** 1 Department of Animal Sciences, College of Agricultural Sciences, Colorado State University, Fort Collins, CO 80523, USA; catie.cramer@colostate.edu (M.C.C.); noa.roman-muniz@colostate.edu (I.N.R.-M.); sofia.thompson@colostate.edu (S.T.); sari.ennis@colostate.edu (S.E.); jordan.marsh@colostate.edu (J.M.); hailey.simpson@colostate.edu (H.S.); ecalaba@rams.colostate.edu (E.C.); 2 Department of Psychology, Colorado State University, Fort Collins, CO 80523, USA; lorann.stallones@colostate.edu (L.S.); elizabeth.kim@colostate.edu (E.K.); 3 Department of Population Health and Pathobiology, College of Veterinary Medicine, North Carolina State University, Raleigh, NC 27607, USA; pairis-garcia@ncsu.edu

**Keywords:** caretakers, euthanasia, mental well-being, moral stress, pigs, training, veterinarians

## Abstract

**Simple Summary:**

Euthanasia is an essential management tool used on livestock operations to alleviate animal suffering. Despite the fact that caretakers who work closely with animals recognize the value of euthanasia, ending an animal’s life remains a difficult task. On swine operations, veterinarians often do not perform day-to-day euthanasia but as animal health and well-being experts, veterinarians should be integral in euthanasia protocol development, training, and execution. Although the importance of euthanasia training is recognized, there is still opportunity within the swine industry to ensure all employees are properly trained. It is evident that there is also a need to provide additional training to veterinarians as integral components of the veterinary school curriculum and continuing education programming. Logistical factors are noted as challenges to proper and timely euthanasia and need to be addressed. Additionally, as the impact that euthanasia can have on caretaker and veterinarian mental well-being becomes more recognized in the livestock industries, it is crucial to incorporate strategies for coping with the moral stress of having to perform euthanasia into training protocols, as currently this is not broadly addressed.

**Abstract:**

Euthanasia is a critical component in swine production and veterinarians play an important role in euthanasia protocol development and training. This study aimed to understand veterinarian involvement in and perspectives on euthanasia on pig farms. An online survey was disseminated both at a pig welfare conference and online via a veterinarian e-newsletter. Twenty-five veterinarians participated in the survey. The majority of respondents indicated that caretakers are the individuals making euthanasia decisions and performing the task (*n* = 17, 68% and 22, 88%, respectively). The majority (22, 88%) of respondents indicated that most of the facilities with which they work have a written euthanasia protocol, and 72% (18) indicated that they assisted in protocol development. Only half of respondents (13, 52%) agreed that “all employees performing euthanasia have been trained adequately”, and 80% (20) identified an interest in delivering more training. Less than half the respondents indicated that strategies for coping with “personal stress” and “emotional wellness” (12, 48%) were included in euthanasia training. While the moral stress of performing euthanasia is recognized, there is opportunity for addressing mental well-being in euthanasia resources. Although preliminary, this study supports the need for further euthanasia training on-farm, involving veterinarians in the process.

## 1. Introduction

Euthanasia is a critical component of on-farm management used as a means to alleviate suffering of diseased or injured pigs that have little chance of recovery [1]. Veterinarians play a crucial role in the development of on-farm euthanasia standard operating procedures that are adopted and implemented on-farm by caretakers [2]. In accordance with the American Veterinary Medical Association (AVMA) *Guidelines for Euthanasia of Animals*, it is the veterinarian’s duty to put the animal’s best interest and welfare at the forefront when making euthanasia decisions and to ensure techniques chosen induce death rapidly and painlessly [3]. Euthanasia is critical to minimize animal suffering when animals are unlikely to return to full health or show improvement even after treatment regimens have been tried [4]. Because veterinarians are central to animal health and welfare, decision-making, training, and recommendations related to euthanasia should fall under their remit.

There are many difficulties associated with bearing the decision to end an animal’s life, even when it is the most humane option for that animal. There are often psychological, emotional, and physical ailments which manifest themselves in caretakers and veterinarians that are tasked with euthanasia responsibilities [5,6]. Considerable research has been conducted, exploring the impacts of performing euthanasia on people who have chosen careers based on their affinity for caring for animals, such as veterinarians, animal shelter workers, livestock owners, and farm employees [7,8]. Reeve et al. [8] found that employees performing euthanasia in animal shelters often develop feelings of misdirected anger that can lead to isolation or discontent. Individuals exhibit a range of reactions to euthanasia, including anger, sadness, fear, guilt, irritability, depression, helplessness, or hopelessness [7,8,9,10], all of which are negative emotions that likely impact long-term psychological and physical well-being. The Reeve et al. [8] study also indicated that employees involved in euthanasia showed significantly higher levels of work stress, stress-induced physical ailments, work-to-family conflict, and dissatisfaction with their work. Individuals that work closely with animals, such as caretakers and veterinarians, form bonds with the animals they care for [11], making performing euthanasia a very difficult task that can have psychological repercussions on those individuals if not addressed [12,13]. Additionally, veterinarians’ main focus is to keep animals healthy, diagnose and treat disease, and promote good animal management, and therefore when euthanasia is determined the best option for an animal, veterinarians may feel a sense of failure [3].

It is important for livestock operations and veterinary practices to have programs, procedures, and support available to caretakers and veterinarians to cope with the moral stress associated with performing euthanasia as part of their job. The quality of an individual’s support network (family, friends, peers, supervisors, etc.) impacts how he/she copes with stress [8]. Relationships with peers and supervisors, in addition to professional psychological services, are integral to effective stress management and these relationships often provide opportunities to discuss stressful situations in a safe and private environment [9]. Other interventions implemented by companies involve counseling resources, job rotation, assistance with job performance, breaks, support groups and meetings, open communication, training, and morale-boosting initiatives [14]. Positive mental health leads to increased job satisfaction and improves one’s ability and willingness to exhibit appropriate and effective behaviors, which increases an organization’s effectiveness [15,16,17]. Studies show that poor mental health increases employee absenteeism and turnover, which diverts organizational resources away from production [16,18,19,20,21]. In summary, the availability of simple resources, support, and stress management tools are highly beneficial to those who must euthanize animals as part of their daily job, and this includes veterinarians.

Previous research evaluating the psychological impact of euthanasia on veterinarians has focused primarily on shelter and companion animal veterinarians. Therefore, there is an increasing need to evaluate the effects of euthanasia on production animal veterinarians. Areas requiring attention in livestock production related to euthanasia are training, written protocols, and clear communication regarding decision-making [22]. Additionally, as veterinary knowledge and guidance is critical in on-farm euthanasia decisions and training, it is essential to have adequate resources available for veterinarians to enhance the experience of caretakers when performing euthanasia. Therefore, the objective of this study was to evaluate veterinarian perceptions of euthanasia training, decision-making, challenges, and considerations for physical and mental health associated with euthanasia duties on swine operations.

## 2. Materials and Methods

The study materials and research plan were approved through the Colorado State University (CSU) Institutional Review Board (#19-9050H) prior to project initiation.

### 2.1. Study Population and Recruitment

The population of interest was veterinarians who routinely work with sows and piglets on swine farms in the United States. Recruitment began at the Pig Welfare Symposium (312 attendees) hosted in Minneapolis, MN in November 2019. Two coauthors attended the conference, hosting a booth and distributing flyers that included study participation details. Additionally, an invitation to participate in an online survey, including a direct survey link, was included in the American Association of Swine Veterinarians’ weekly electronic newsletter (1585 subscribers). The survey information was distributed in February 2020 and was included in the e-newsletter for two consecutive weeks. Respondents were offered a $25 gift card for participation. Those who elected to receive the gift card were asked to leave an email address. All responses remained anonymous and no identifying information, including email address, was associated with responses given. The only forced response question in the survey was the participation consent; all other questions were optional.

### 2.2. Survey Development and Content

The survey was developed by a multidisciplinary team with expertise in animal sciences, veterinary medicine, public health, and epidemiology, and well-versed in survey development and administration. Some questions were modeled from a similar survey conducted by [23] with dairy veterinarians. The survey was developed in Qualtrics software (Qualtrics, Provo, UT, USA) and, prior to survey distribution, all questions were independently reviewed by the coauthors to ensure content validity. Additionally, the survey was tested by a group of graduate students within the Department of Animal Sciences at CSU to ensure functionality and clarity.

The entire survey included a total of 67 questions and was intended to take less than 30 min to complete. These questions represented a portion of a larger survey exploring perspectives of managers and caretakers on swine operations and therefore a branching method was used in the survey when respondents indicated their role as a veterinarian. The entire survey is provided as supplementary material. The categories of questions included: demographics and background, euthanasia methods used, role in euthanasia decision-making, training delivery and methods, perspectives of euthanasia, support networks, and knowledge and availability of wellness programs. A variety of question types were utilized in the survey, including dichotomous, multiple choice, Likert scale, and open-ended questions.

### 2.3. Statistical Analysis

Once the survey was closed, all data were exported to a Microsoft Excel (Microsoft Corporation, Redmond, WA) spreadsheet. Two individuals independently reviewed the data for entry errors and completeness. Twenty-five surveys were completed. All surveys were >80% complete and therefore included in the analysis. Some respondents either declined to answer or provided no answer for various questions; these categories were noted in all data summaries where applicable. Descriptive statistics were tabulated for all variables of interest. Due to the relatively small sample size, no additional statistical analysis was completed.

## 3. Results

Twenty-five surveys were returned. There were 312 attendees at the Pig Welfare Symposium. The American Association of Swine Veterinarians (AASV) electronic newsletter was distributed to 1585 subscribers. It is estimated that the response rate was at least 1.3%, as not all the conference attendees were veterinarians, and thus not all were eligible to participate in the survey. Due to this relatively low response rate, we will consider the results preliminary, warranting further exploration with a larger sample population.

### 3.1. Demographics

The demographics of the respondents are summarized in Table 1. Two-thirds of the survey respondents (*n* = 16, 64%) identified as female. Most of the respondents were from the Midwest (17, 68%; U.S. regions as defined in O’Connor [24]). The majority of respondents identified as non-Hispanic or non-Latino (22, 88%) and indicated English as their native language (20, 80%). Approximately one-third of respondents indicated they either served “multiple companies with several site locations” (9, 36%) or “one company with several site locations” (9, 36%). Slightly less than half of respondents (11, 44%) indicated that they had previous employment with other swine operations within the United States.

### 3.2. Veterinarian Involvement with Euthanasia Responsibilities

The majority of respondents indicated that they “work with pigs often” (22, 88%). The other 12% (three) identified that they “work with pigs occasionally”. All respondents except one indicated that they had euthanized at least one pig in the past 12 months. Respondents were asked to identify the most common method they used for euthanizing sows and piglets (Table 2). The majority of respondents (16, 64%) indicated that captive bolt was the most common euthanasia method they used for sows; however, the following were also identified: injection, gunshot, electrocution, and blunt force trauma. When asked about piglets, a third of respondents (9, 36%) indicated that blunt force trauma was the most common method of euthanasia they used; however, the following methods were also provided: carbon dioxide, non-penetrating captive bolt, injection, and electrocution. Respondents were asked an open-ended question regarding “what determines which euthanasia method is used”. Many respondents provided multiple answers, including: animal characteristics (e.g., size and age of the pig), the written protocol at the facility, industry guidelines and policies (e.g., National Pork Board (NPB), AASV), the condition of the animal (i.e., is it suffering?), the safety of the method for employees, and the humaneness of the method.

Although the majority of respondents (20, 80%) indicated that they had a veterinary–client–patient relationship (VCPR) with the operations they work with, 12% (three) indicated they did not have a VCPR and 8% (two) declined to answer. Respondents were asked if the swine facilities where they served as a veterinarian had a written protocol for euthanasia, to which the majority indicated “yes” (22, 88%). Although still a majority (18, 72%), fewer respondents indicated that they were involved in authoring the euthanasia protocol; the remaining respondents indicated that either they were not involved in the creation of the protocol (4, 16%) or answered “not applicable” (3, 12%).

**Figure 1:**
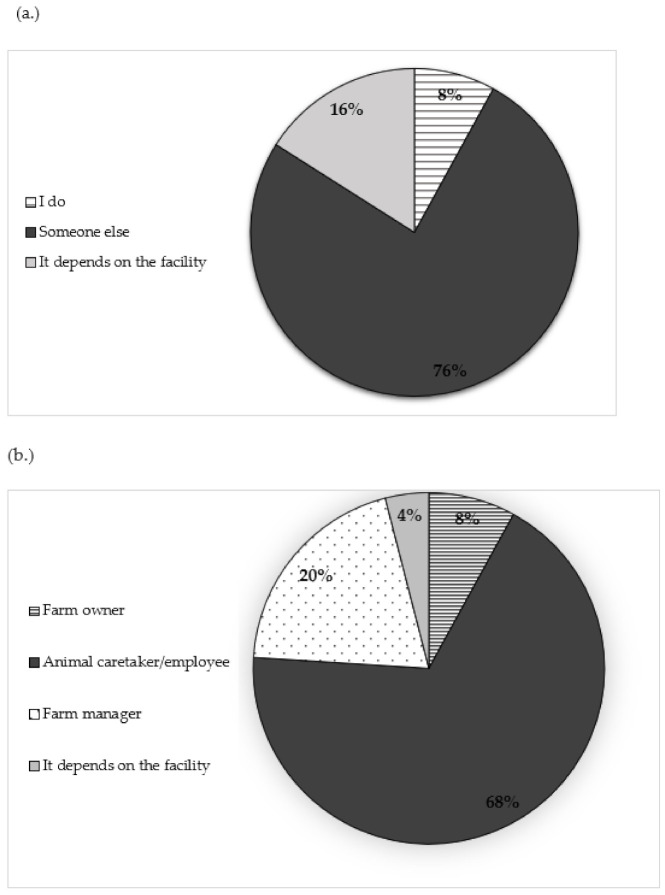
Respondents were asked about decision-making around euthanasia (*n* = 25). (**a**) Respondents were asked “On most of the facilities where you serve as a veterinarian, who makes the decision to euthanize?” The options provided for selection were: I do, someone else, it depends on the facility. (**b**) Respondents were asked “If someone else makes the decision to euthanize, what is this person’s role?” The options provided for selection were: farm owner, farm manager, animal caretaker/employee, it depends on the facility, or decline to answer.

**Figure 2:**
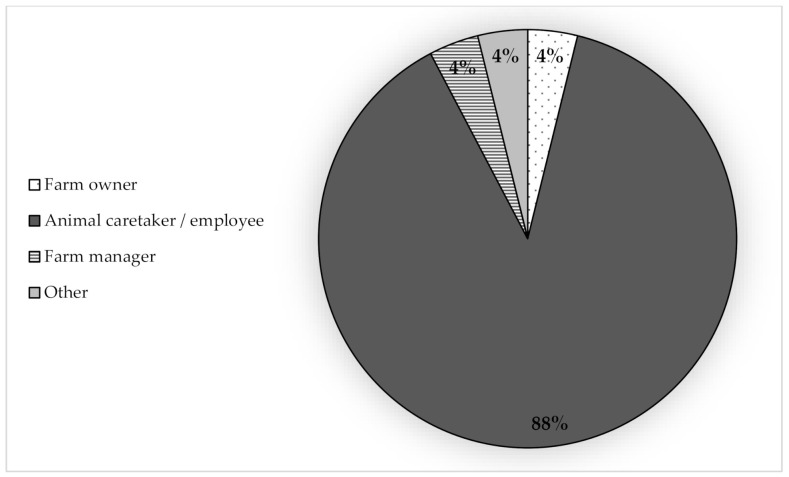
Respondents were asked “If someone else performs most of the euthanasias, what is this person’s role?” (*n* = 25). The options provided for selection were: farm owner, farm manager, animal caretaker/employee, other, or decline to answer.

Respondents were asked several questions regarding euthanasia decision-making. Figure 1a,b illustrates the different roles of individuals involved in euthanasia decision-making on-farm. Three-quarters of respondents (19, 76%) indicated that someone other than the respondents themselves (i.e., the veterinarian) makes euthanasia decisions (Figure 1a). When asked to identify the person’s role who makes euthanasia decisions, 68% (17) of respondents indicated that animal caretakers/employees are making euthanasia decisions (Figure 1b). Managers and owners were also identified as making decisions about euthanasia (5, 20% and 2, 8%, respectively). Additionally, the majority of respondents (20, 80%) indicated that clients “sometimes/a few cases” consulted them before euthanizing a pig (Table 3). Only one respondent (4%) indicated that they were “always/every case” consulted. When asked about the role of individuals who perform euthanasia, 88% (22) of respondents indicated that animal caretakers/employees perform euthanasia on the farms they work with (Figure 2).

### 3.3. Training

**Figure 3:**
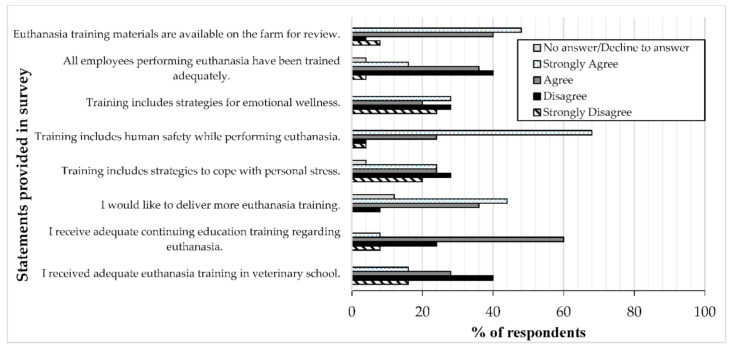
Respondents (*n* = 25) were asked to indicate their level of agreement with various statements related to euthanasia training. The options available to choose from were: Decline to answer, Strongly agree, Agree, Disagree, and Strongly Disagree. Some respondents did not answer the question.

Respondents were asked an open-ended question regarding how they deliver euthanasia training on-farm. Many respondents provided a combination of methods, including in-person training, presentations, manuals, videos, and hands-on learning. Almost all respondents provided a description that suggested an in-person component to euthanasia training. Language used to describe in-person components included: on-site, one-on-one practical training, on-farm, hands-on, and in-person. Respondents were provided with multiple statements related to training and asked to indicate their level of agreement (Figure 3). Although approximately half of respondents (13, 52%) either agreed or strongly agreed that “all employees performing euthanasia have been trained adequately”, 44% (11) disagreed or strongly disagreed with this statement. Most respondents (22, 88%) agreed or strongly agreed that “training materials are available on the farm for review”. The vast majority of respondents (23, 92%) agreed or strongly agreed that “training includes human safety while performing euthanasia”, whereas slightly less than half of respondents indicated agreement with statements asking about the inclusion of “emotional wellness” and “personal stress” (12, 48% for both statements). When asked about their own training, 56% (14) disagreed or strongly disagreed that they “received adequate euthanasia training in veterinary school”. Approximately one-third of respondents (8, 32%) indicated that they disagreed or strongly disagreed that they “received adequate continuing education training regarding euthanasia”. Additionally, the majority of respondents (20, 80%) agreed or strongly agreed that they “would like to deliver more euthanasia training”.

### 3.4. Wellness Programs

Approximately half of respondents (12, 48%) indicated that there are “programs to promote worker health” at the facilities with which they work. Only one respondent (4%) indicated that there were “mental health evaluations” and 36% (nine) indicated there were “employee check-ins with a supervisor or administrator”. When asked for further clarification on the details of these programs and evaluations, some of the open-ended responses included: workplace safety programs, annual evaluations of work, overall wellness exams, monthly wellness topics, programs through health insurance, and counseling through employee assistance programs. Approximately half of respondents (12, 48%) knew what mental health care resources were available in their communities; 8% (two) of individuals indicated that this question was “not applicable”.

Only one respondent (4%) disagreed about feeling they could communicate with supervisors if feeling uncomfortable performing euthanasia; five (20%) respondents did not provide an answer to the statement. When asked about the number of friends, relatives, and work peers that respondents can communicate with regarding their feelings about euthanasia, the reported ranges were 0 to 25, 0 to 20, and 0 to 100, respectively.

### 3.5. Perceptions and Communication about Euthanasia

**Figure 4:**
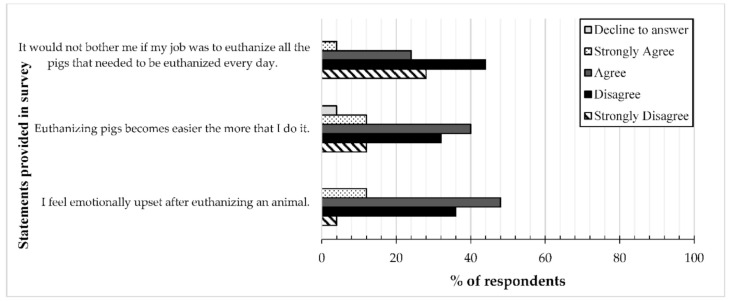
Respondents (*n* = 25) were asked to indicate their level of agreement with various statements related to perceptions about euthanasia. The options available to choose from were: Decline to answer, Strongly Agree, Agree, Disagree, and Strongly Disagree.

All respondents (25, 100%) agreed or strongly agreed with the following statements: “euthanasia is a humane way to end suffering”, “it is more humane to euthanize animals that are suffering than to let them die naturally”, “there are often good reasons for euthanizing pigs”, and “the euthanasia process on farm is necessary”. Only one individual (4%) responded that they did not “have enough experience and knowledge to know when to euthanize a pig”. Approximately two-thirds (15, 60%) of respondents agreed or strongly agreed that they “feel emotionally upset after euthanizing an animal” and 52% (13) indicated agreement with the statement “euthanizing pigs becomes easier the more I do it” (Figure 4). Less than a third (7, 28%) of respondents agreed or strongly agreed that “it would not bother me if my job was to euthanize all the pigs that needed to be euthanized every day” (Figure 4).

Respondents were asked if there was any aspect of the euthanasia process that bothers or distresses them. A number of respondents indicated that death is itself just difficult to deal with because it is complicated and emotional but there is comfort knowing it is ending animal suffering. For instance, one respondent shared: “it is always bothersome to euthanize an animal; however, it is comforting to know that, when performed, it is done to end an animal’s suffering in a humane way so that the animal feels no pain”. Another common response was that it is distressing when euthanasia is performed improperly, whether that is due to lack of caring on the part of the person performing euthanasia, or inappropriate or ill-maintained equipment. When asked if there were any additional thoughts to share, only a few respondents shared some thoughts, with one in particular related to perspectives about euthanasia: “Euthanasia shouldn’t become “easier” the more it’s done, because you should still feel empathy for the animal while recognizing that they should not have to endure prolonged suffering. Euthanasia will always have an emotional burden attached to it, because if it became “easy” then there is no humanity/empathy involved in respect to the animals’ life”.

### 3.6. Challenges, Resources, and Needs

Respondents were asked where they currently get most of their information on euthanasia methods. Respondents mentioned all of the industry organizations that have relevant information regarding euthanasia, including: AASV, AVMA, NPB (generally and with specific mention of the Pork Quality Assurance program), the World Organization for Animal Health, North American Meat Institute, and the National Pork Producers’ Council. Other responses included some regional-specific resources, scientific literature, and conferences. Respondents were asked an open-ended question to indicate what resources or materials regarding euthanasia would be beneficial to them as veterinarians. Several individuals indicated that they have enough material and access to resources. The majority provided suggestions with general thoughts related to alternate methods of euthanasia for certain animal types (e.g., piglets, large pigs), detailed guides specific to certain sites, training materials for employees that are concise and relevant to them, and options for mass euthanasia.

Respondents were asked to share some unexpected challenges with euthanasia at the operations they work with. Many respondents mentioned equipment and operator errors; comments were shared regarding lack of equipment maintenance, malfunctioning equipment, individuals performing the job incorrectly, and general equipment needs. Additionally, staff turnover and having inconsistent protocols were mentioned as challenges. Timely euthanasia was mentioned as a challenge by several respondents; interestingly, a couple of comments identified that the issue with timeliness often came from an “overabundance of compassion” and a “desire to keep the pigs alive”. “Risks to mental health” and “new caretakers’ resistance and uncomfortable reactions to the process” were also included in responses.

## 4. Discussion

The survey population in this study differed from published reports of food animal veterinarian demographics in the United States [25]. Food animal-exclusive veterinary medicine is dominated by men (77%), whereas women constituted the majority (64%) of the respondents in this survey [25]. A 2012 AVMA Workforce Survey reported that 54 years was the median age of the survey population, an older population than the current study (median = 35 years) [26]. The upper Midwest region of the United States, including states such as Iowa, Illinois, Minnesota, and Michigan, contain a higher concentration of food animal-exclusive veterinarians, paralleling the demographics of the respondents from this survey [27]. These differences in demographics and the relatively small sample size should be taken into account when considering the results from this study.

The objective of this study was to explore the perspectives of swine veterinarians on euthanasia with specific interest in euthanasia decision-making, training, impacts on mental wellness, and challenges. It is evident from responses across the Table S1 survey questions that while the participating veterinarians affirmed the importance of euthanasia and its essential role in swine management, they also recognized the difficulties with performing the task. Challenges identified by veterinarians ranged from having well-maintained equipment to the emotional burden of having to take an animal’s life even if the best option for the animal. These feelings are not unique to these study respondents and have been reported repeatedly in research conducted in a variety of animal industries [5,7,23,28,29,30].

In the current study, over half of the respondents noted feeling emotionally upset after euthanizing animals and slightly less agreed that euthanasia becomes easier the more you do it. A quote shared earlier identified that in theory euthanasia should never get easier or the empathy needed would be diminished. That being said, individuals must balance the emotional strain of maintaining care for animals with the need to protect one’s own mental wellness. In one of the foundational studies of livestock worker perspectives of euthanasia, Matthis [31] found that despite understanding the importance of euthanasia to swine welfare, nearly half of the participants would be content to not have to perform euthanasia. Interestingly, despite the noted negative emotions associated with performing euthanasia, approximately a third of respondents in the current study indicated they would not mind if performing euthanasia was solely their responsibility. A follow-up question was not asked to determine why respondents felt this way but perhaps individuals, veterinarians and caretakers alike, take on the extra burden of performing euthanasia because they know they will do it correctly. Campler et al. [30] reported that swine caretakers characterized as “confident and empathetic”, on average disagreed that if given the choice they would “prefer someone else to euthanize pigs rather than myself”, demonstrating a desire to do what is right for the animal even if it causes them moral stress. Individuals that care for animals can often find consolation when reminding themselves that euthanasia is humane, important, and necessary for the animals in need [7,9,32,33].

When asked about challenges with euthanasia on-farm, many respondents noted logistical issues related to improperly maintained equipment, the need for additional resources, alternate methods, and challenges with certain animal types. Mullins et al. [1] similarly identified logistical challenges as common barriers to timely euthanasia, including equipment availability and maintenance. In a thematic analysis of dairy veterinarian focus groups, Wagner et al. [23] reported logistical factors, including financial/economical, protocols/procedures/guidelines, client/operation/farm size, carcass disposal, time/labor/space, equipment, and ownership, as common barriers to timely euthanasia on dairies. All these logistical factors are fixable over time and can be addressed through standard operating procedures, training, etc. However, many of these issues are interconnected and the presence of multiple logistical factors compounds challenges faced by veterinarians. Although not a directly asked question in this survey, some respondents identified mass euthanasia as a challenge. This has been mentioned in other studies as well, specifically related to porcine epidemic diarrhea virus (PEDv) [1], Johne’s disease [34], and foot and mouth disease [35]. Considering some of the recent challenges experienced with the COVID-19 pandemic, veterinarian and caretaker perspectives of mass euthanasia and depopulation is an area worthy of further exploration.

Discussions regarding euthanasia protocols and decision-making should be part of the established duties of the veterinarian on-farm [36]. A VCPR is the basis for interaction between veterinarians and their clients and patients. The importance, necessity, and nature of VCPRs are laid out in multiple industry resource documents, including the AVMA [37] and the NPB Pork Quality Assurance (PQA) Plus^®^ program [38]. Additionally, having a valid VCPR, verified by reviewing dated written material such as veterinary feed directives, prescription labels, or a veterinarian letter confirming the relationship, is an audit criterion within the Common Swine Industry Audit (CSIA) [39]. National swine industry data indicated that 98.2% of participating swine operations acknowledged having a VCPR [40]. In the current study, although the majority, only 80% of veterinarians noted having a VCPR with the facilities they currently work with. The remaining respondents either did not have one or declined to answer. Although the sample size in this study was small, it is worth noting this result as a VCPR is foundational to providing veterinary services.

Veterinarians have differing relationships with the facilities for which they provide services. Within the swine industry, private practice veterinarians commonly serve swine operations, compared to, for instance, the poultry industry, which relies more upon company-employed veterinarians [41]. Although reported in the context of antimicrobial use and stewardship, the 2017 National Animal Health Monitoring System (NAHMS) report indicated that, of the participating swine operations, only 15.4% of sites were visited by an on-staff or company veterinarian in the preceding months, compared with 55.8% being visited by a local veterinary practitioner or a consulting or second-opinion veterinarian [40]. Most respondents in the current study either worked with multiple companies with several site locations or one company with several site locations. The type of relationship between a veterinarian and the swine operation may somewhat determine the balance between primary animal care responsibilities (such as performing euthanasia) and other more specialized veterinary tasks (such as development of treatment and euthanasia protocols), and generally food animal veterinarians are not present on-farm frequently enough to perform tasks such as euthanasia [36]. In the current study, the majority of the respondents were not the individuals making euthanasia decisions or performing euthanasia but were sometimes consulted on a few cases. Wagner et al. [23] reported similar findings with dairy veterinarians; 68.9% of study participants indicated that someone other than themselves performed the majority of euthanasias on the dairy operations where they served as veterinarians. A study focusing on dairy producer perspectives on euthanasia reported that approximately a third of participants indicated consulting a veterinarian on euthanasia decisions, substantially more than was reported in the current study [2].

Even though veterinarians did not make day-to-day decisions on euthanasia in this study, the majority (72%) did participate in the development of the written protocol for euthanasia on-farm, and most of the farms the respondents worked with had protocols. Having written procedures for euthanasia is critical, particularly because the impacts of performing euthanasia incorrectly can have significant consequences for human and animal welfare [22,42]. Despite the significance of this task, in addition to the fact that having a written euthanasia protocol is a requirement in the CSIA [39] and is outlined in the PQA Plus^®^ program [38], there are still swine operations that do not have written protocols for euthanasia. Lack of written euthanasia protocols has also been identified in the dairy industry; Wagner et al. [23] reported greater than 40% of their study participants indicated that most of the facilities respondents served did not have a written protocol for euthanasia. Although a need, the mere existence of the protocol is not enough; the protocol must also be accessible to the caretakers that it is relevant to, both by being visually present at the operation and also provided in the native language of those performing the job [23]. In the current study, the majority of respondents did indicate that training materials were available on-farm for review when needed.

The swine industry has many resource documents related to proper techniques for performing euthanasia, including information in the PQA Plus^®^ program [38], the NPB and AASV on-farm euthanasia recommendations [4], and euthanasia guidelines set forth by the AVMA [3]. When asked what resources the survey respondents used to determine method of euthanasia, many of these aforementioned organizations and materials were identified, indicating that these resources are likely readily available to veterinarians. Past studies exploring caretaker and veterinary perspectives regarding euthanasia in various livestock industries have all identified the importance of euthanasia training [2,22,23,27]. Training, in general, is important to employee success [43] and positive interactions on-farm between animals and the caretakers, particularly when talking about a challenging task such as euthanasia, and can truly impact caretakers’ job satisfaction [44]. Despite this recognition and the availability of materials, there are still opportunities for training within the swine industry. In the current study, only half of the respondents felt that caretakers were adequately trained on euthanasia, despite the fact that the majority indicated that on the farms they worked with there was a written euthanasia protocol and training materials were available on-farm for review. Similarly, McGee et al. [45] also reported that half of participating swine caretakers had received euthanasia training in the past year. McGee et al. [45] indicated that almost a third of their survey participants would like more training; this has been similarly shared by dairy workers who indicated a general desire for more in-person, on-the-job training [46]. For a task that has critical impacts on both human and animal welfare, training is certainly an area of needed attention.

Training on production facilities can come in many different forms, such as presentations with multimedia (i.e., slides and videos), review of protocols and resource documents, or in-person demonstrations and hands-on experience [43]. In the current study, the majority of respondents indicated that they perform some type of in-person training on the farms with which they work. In line with the findings of Wagner et al. [23], indicating that dairy cattle veterinarians would like more opportunities for interactive euthanasia training with caretakers, the vast majority of the respondents in the current study wanted to be more involved with euthanasia training on-farm. McGee et al. [45] indicated that swine caretakers preferred on-farm euthanasia training with classroom follow up, including written materials and video. Campler et al. [47] have explored the use of an interactive, computer-based euthanasia training program for swine caretakers and identified a self-reported improvement in knowledge post-training. There is a clear opportunity across livestock industries to further engage veterinarians in preparing caretakers for both decision-making and performance of this critical management task using multimodal delivery methods.

Discussions about euthanasia training usually revolve around the animal caretakers as they are the individuals more commonly performing the task. In the current survey, respondents were asked to comment on their own euthanasia training and the shared message was that although the majority felt they had the experience and knowledge to perform the task, they did not necessarily receive enough euthanasia training in veterinary school or through continuing education. A 2011 survey, including 21 AVMA-accredited veterinary medical colleges in the United States, Canada, and the Caribbean, found that only 10% of schools offered euthanasia and quality-of-life content and training [48]. Euthanasia training was generally included as a core topic, rather than elective credits, but students at the participating institutions only received an average of 4.4 h of training on this topic while in veterinary school [48]. One study suggested that veterinary schools might only be able to provide 15 h of euthanasia and end-of-life training maximum due to the already dense nature of the curriculum [49]. Dickinson et al. [49] proposed increased support of continuing education and symposiums by veterinary schools to channel current and graduated veterinary students towards needed euthanasia education. The only euthanasia training veterinary students might obtain is from their clinical rotations, should cases requiring consideration of euthanasia occur; additionally, it is not known how much livestock-specific training is available. Veterinary-specific euthanasia training, pre- and post-veterinary school, is an area in need of further development.

Interestingly, despite the strong message across studies that euthanasia training is needed on livestock production facilities [2,29,43,47], in all the recommendations there is rarely a direct suggestion that the euthanasia training should include mental health awareness. As has been mentioned, even if individuals appreciate the importance of euthanasia as a tool to alleviate animal suffering, which most do, actually performing the job can be stressful. In the current study, the majority of respondents indicated that human safety components were included in training, as they should be, but fewer indicated that strategies for coping with personal stress and emotional wellness were part of on-farm training. In this study, only half of the respondents indicated awareness of mental health resources within their communities. Half also indicated that there were programs to promote worker health at the facilities with which they worked but only a third of the facilities actually had employee check-ins. This survey did not provide a specific definition of these items but the intent was to gain a preliminary understanding of existing resources and avenues of communication that may be capitalized upon to help alleviate some of the stress associated with euthanasia. One interesting comment in one of the open-ended questions was the concern about new caretakers’ initial negative reactions to the euthanasia process. Reeve et al. [7] found that “turning point” events can significantly impact individuals’ future feelings towards euthanasia and cited the first euthanasia experience as one of these potential turning point events. It is important to provide individuals with the technical skills and confidence to perform euthanasia but also the understanding of why euthanasia is important and how to cope with the associated stress. The availability of simple resources, support, and stress management tools are highly beneficial to those who participate in both performing and making euthanasia decisions. Some examples of effective resources to address the mental stress of euthanasia utilized in animal shelters include counseling resources, job rotation, assistance with job performance, breaks, support groups and meetings, open communication, training, and morale-boosting initiatives [14]. Future work should explore what type of strategies can be implemented on swine operations around the topic of euthanasia to promote caretaker and veterinarian mental well-being.

## 5. Conclusions

The preliminary results presented in this study suggest an opportunity for further involvement of swine veterinarians in on-farm training of animal caretakers. Although veterinarians may not be the individuals making the decisions on euthanasia and performing the task, they are the animal health experts that can provide guidance on protocols, decision trees, and training. Although the majority of respondents noted that written protocols for euthanasia were present, it is essential to make sure that these protocols are understood and accessible to all caretakers. In this study population, there was a clear desire to deliver more training. Efforts should be focused on providing more training opportunities for both veterinarians and animal caretakers. Additionally, strategies to deal with the mental well-being impacts of performing euthanasia should be included into training and other management frameworks to provide support for those who make decisions about and perform euthanasia as part of their job. Practical suggestions to improve managing euthanasia, euthanasia training, and support networks that offer effective coping mechanisms for euthanasia personnel are a critical need in the industry. As noted, the data presented are preliminary and further research in this area is warranted.

## Figures and Tables

**Table 1:** Demographics of survey respondents (*n* = 25).

Gender	
Male	36 (9)
Female	64 (16)
**Age (years)**	
Average	38.6
Range	27 to 62
**Location of current residence ^1^**	
Midwest	68 (17)
Southwest	4 (1)
West	4 (1)
Southeast	4 (1)
Outside of North America	20 (5)
**Ethnicity**	
Hispanic or Latino	8 (2)
Non-Hispanic or Non-Latino	88 (22)
Decline to answer	4 (1)
**Native language**	
English	80 (20)
Spanish	4 (1)
Portuguese	4 (1)
Polish	4 (1)
Dutch	4 (1)
Korean	4 (1)
**Years with current employer ^2^ (years)**	
Average	5.9
Range	0.6 to 14.7
**Role as a swine veterinarian**	
Multiple companies with several site locations	36 (9)
One company with one site location	8 (2)
One company with several site locations	36 (9)
Other ^3^	16 (4)
Decline to answer	4 (1)
**Previous employment with other US swine operations**	
Yes	44 (11)
No	40 (10)
Decline to answer	16 (4)

^1^ Regions were defined as quoted by O’Connor [24]. ^2^ Two respondents did not answer (*n* = 23). ^3^ Respondents who selected “Other” indicated the following: researcher/veterinarian at university farm, part of production well-being team, works with independent producers in the show pig industry, and technical services veterinarian.

**Table 2:** Respondent answers when asked to indicate “the most common euthanasia method you use” for sows and piglets (*n* = 25). Respondents were asked to fill in the method; no options were provided.

Method of Euthanasia	Respondents % (*n*)
*Sows*	
Captive bolt	64 (16)
Injection	12 (3)
Gunshot	8 (2)
Electrocution	8 (2)
Blunt force trauma	4 (1)
No response	4 (1)
	
*Piglets*	
Blunt force trauma	36 (9)
Carbon dioxide	28 (7)
Non-penetrating captive bolt	20 (5)
Injection	8 (2)
Electrocution	4 (1)
No response	4 (1)

**Table 3:** Respondents were asked “In the past 12 months, how often did your clients consult you before euthanizing a pig?” (*n* = 25).

Frequency of Consultation	Respondents % (*n*)
Always/every case	4 (1)
Often/most cases	4 (1)
Sometimes/a few cases	80 (20)
Never/no cases	12 (3)

## Supplementary Materials

The following are available online at https://www.mdpi.com/2076-2615/10/10/1919/s1, Table S1: Survey Questions.
