# Supplementary material for: Preliminary Exploration of Swine Veterinarian Perspectives of On-Farm Euthanasia
Source: Animals (Basel). 2020 Oct 19;10(10):1919. doi: 10.3390/ani10101919 (PMC7650797; doi:10.3390/ani10101919)
Supplement: Supplementary file 1 [file animals-10-01919-s001.zip › animals-969956-supplementary.docx]

**Supplementary Material**

Table S1: Survey Questions

If your primary language is English, please select English. Si su idioma principal es el Español, seleccione Español.

1. Please type the complete name of the state in which you reside :
2. How would you describe your current role in the swine industry?
   1. Owner – works with pigs occasionally
   2. Owner – works with pigs often
   3. Manager – works with pigs occasionally
   4. Manager – works with pigs often
   5. Caretaker – works with pigs occasionally
   6. Caretaker – works with pigs often
   7. Veterinarian – works with pigs occasionally
   8. Veterinarian – works with pigs often
   9. Decline to answer question
3. In the past 12 months, have you euthanized any pigs?
   1. Yes
   2. No
   3. Decline to answer
4. What is the most common euthanasia method you use for sows?
5. What is the most common euthanasia method you use for piglets?
6. Which of the following best describes your role as a swine veterinarian?
   1. One company with one site location
   2. One company with several site locations
   3. Multiple companies with several site locations
   4. Other
   5. Decline to answer
7. Do you have a VCPR with the operations you work with?
   1. Yes
   2. No
   3. Decline to answer
8. If someone else performs most of the euthanasias, what is this person's role?
   1. Farm owner
   2. Farm manager
   3. Animal caretaker / employee
   4. Other, please specify:
   5. Decline to answer
9. On most of the swine facilities where you serve as the veterinarian, who makes the decision to euthanize?
   1. I do
   2. Someone else
   3. It depends on the facility. Please explain:
   4. Decline to answer
10. If someone else makes the decision to euthanasia, what is this person's role? - Selected Choice
    1. Farm owner
    2. Farm manager
    3. Animal caretaker / employee
    4. It depends on the facility please specify:
    5. Decline to answer
11. Do most swine facilities where you serve as a veterinarian have a written protocol for euthanasia?
    1. Yes
    2. No
    3. Decline to answer
12. In your role as the facility's veterinarian, were you involved in the making of the euthanasia protocol?
    1. Yes
    2. No
    3. Not applicable
    4. Decline to answer
13. In the past 12 months, how often did your clients consult you before euthanizing a pig?
    1. Always/ every case
    2. Often/ most cases
    3. Sometimes/ a few cases
    4. Never/ no cases
14. What determines which euthanasia method is used?
15. What are some unexpected challenges with euthanasia at the operations you work with?
16. What resources or materials regarding euthanasia would be beneficial to you as a veterinarian?
17. Where do you get most of your information on euthanasia methods, new information, etc?
18. In general, how do you deliver training on euthanasia?
19. I received adequate euthanasia training in veterinary school.
    1. Strongly disagree
    2. Disagree
    3. Agree
    4. Strongly agree
    5. Decline to answer
    6. Not applicable
20. I receive adequate continuing education training regarding euthanasia.
    1. Strongly disagree
    2. Disagree
    3. Agree
    4. Strongly agree
    5. Decline to answer
    6. Not applicable
21. I would like to deliver more euthanasia training.
    1. Strongly disagree
    2. Disagree
    3. Agree
    4. Strongly agree
    5. Decline to answer
    6. Not applicable
22. Training includes strategies to cope with personal stress.
    1. Strongly disagree
    2. Disagree
    3. Agree
    4. Strongly agree
    5. Decline to answer
    6. Not applicable
23. Training includes human safety while performing euthanasia.
    1. Strongly disagree
    2. Disagree
    3. Agree
    4. Strongly agree
    5. Decline to answer
    6. Not applicable
24. Training includes strategies for emotional wellness.
    1. Strongly disagree
    2. Disagree
    3. Agree
    4. Strongly agree
    5. Decline to answer
    6. Not applicable
25. All employees performing euthanasia have been trained adequately.
    1. Strongly disagree
    2. Disagree
    3. Agree
    4. Strongly agree
    5. Decline to answer
    6. Not applicable
26. Euthanasia training materials are available on the farm for review.
    1. Strongly disagree
    2. Disagree
    3. Agree
    4. Strongly agree
    5. Decline to answer
    6. Not applicable
27. At work:

|  |  | | | Frequency | | Additional notes regarding answer |
| --- | --- | --- | --- | --- | --- | --- |
|  | Yes | No | Decline to answer | How many take place per year? | Decline to answer | Please add any clarifying information if necessary: |
| Are there programs to promote worker health? |  |  |  |  |  |  |
| Are there any mental health evaluations? |  |  |  |  |  |  |
| Are there employee check-ins with a supervisor or administrator? |  |  |  |  |  |  |

1. If you have answered yes to any of the questions above, please explain the details of any program, evaluation or check-ins you may have at your workplace. If no, type N/A or leave blank.
2. Do you feel that these programs, evaluations or check-ins benefit your overall happiness in your job? Please, explain. If this does not apply to you, type N/A or leave blank.
3. I have received guidance/advice on how to manage the stress in my workplace.
   1. Strongly disagree
   2. Disagree
   3. Agree
   4. Strongly agree
   5. Decline to answer
   6. Not applicable
4. My workplace has access to programs and/or training to help me adequately deal with my work responsibilities.
   1. Strongly disagree
   2. Disagree
   3. Agree
   4. Strongly agree
   5. Decline to answer
   6. Not applicable
5. I feel physically safe and protected at work while preforming euthanasia.
   1. Strongly disagree
   2. Disagree
   3. Agree
   4. Strongly agree
   5. Decline to answer
   6. Not applicable
6. My job has adequate programs to help me cope with my job.
   1. Strongly disagree
   2. Disagree
   3. Agree
   4. Strongly agree
   5. Decline to answer
   6. Not applicable
7. I am satisfied with my current job.
   1. Strongly disagree
   2. Disagree
   3. Agree
   4. Strongly agree
   5. Decline to answer
   6. Not applicable
8. I feel supported by my peers in the workplace.
   1. Strongly disagree
   2. Disagree
   3. Agree
   4. Strongly agree
   5. Decline to answer
   6. Not applicable
9. Please rate how your workplace values the mental health of employees.
   1. Not valued
   2. Somewhat valued
   3. Valued
   4. Very valued
   5. Decline to answer
   6. Not applicable
10. The following question relates to your employment. Please rate your experience with each topic by checking the appropriate box:
11. I believe that euthanasia is a humane way to end animal suffering.
    1. Strongly disagree
    2. Disagree
    3. Agree
    4. Strongly agree
    5. Decline to answer
    6. Not applicable
12. It is more humane to euthanize animals that are suffering than to let them die naturally.
    1. Strongly disagree
    2. Disagree
    3. Agree
    4. Strongly agree
    5. Decline to answer
    6. Not applicable
13. I feel as though the euthanasia process on the farm is necessary.
    1. Strongly disagree
    2. Disagree
    3. Agree
    4. Strongly agree
    5. Decline to answer
    6. Not applicable
14. I have enough experience and knowledge to know when to euthanize a pig.
    1. Strongly disagree
    2. Disagree
    3. Agree
    4. Strongly agree
    5. Decline to answer
    6. Not applicable
15. I feel as though there are often good reasons for euthanizing pigs.
    1. Strongly disagree
    2. Disagree
    3. Agree
    4. Strongly agree
    5. Decline to answer
    6. Not applicable
16. I feel emotionally upset after euthanizing an animal.
    1. Strongly disagree
    2. Disagree
    3. Agree
    4. Strongly agree
    5. Decline to answer
    6. Not applicable
17. Euthanizing pigs becomes easier the more that I do it.
    1. Strongly disagree
    2. Disagree
    3. Agree
    4. Strongly agree
    5. Decline to answer
    6. Not applicable
18. It would not bother me if my job was to euthanize all the pigs that needed to be euthanized every day.
    1. Strongly disagree
    2. Disagree
    3. Agree
    4. Strongly agree
    5. Decline to answer
    6. Not applicable
19. Is there any aspect of the euthanasia process that bothers or distresses you? Please explain.
20. Is there anything else you would like to share about your role in performing euthanasia?
21. Is there anything else you would like to share about euthanasia?
22. I feel as though I can communicate with my supervisors if I feel uncomfortable performing euthanasia.
    1. Strongly disagree
    2. Disagree
    3. Agree
    4. Strongly agree
    5. Decline to answer
    6. Not applicable
23. I am aware of proper channels to communicate issues to management.
    1. Strongly disagree
    2. Disagree
    3. Agree
    4. Strongly agree
    5. Decline to answer
    6. Not applicable
24. My supervisors aim to promote a safe and encouraging work environment.
    1. Strongly disagree
    2. Disagree
    3. Agree
    4. Strongly agree
    5. Decline to answer
    6. Not applicable
25. I feel as though my supervisors acknowledge concerns that I may have.
    1. Strongly disagree
    2. Disagree
    3. Agree
    4. Strongly agree
    5. Decline to answer
    6. Not applicable
26. My supervisor takes an interest in my professional development and/or job performance.
    1. Strongly disagree
    2. Disagree
    3. Agree
    4. Strongly agree
    5. Decline to answer
    6. Not applicable
27. My supervisor takes an interest in my over-all well-being.
    1. Strongly disagree
    2. Disagree
    3. Agree
    4. Strongly agree
    5. Decline to answer
    6. Not applicable
28. How many people do you talk to about your work?
    1. Relatives:
    2. Friends:
    3. Work peers:
    4. Other:
    5. Not applicable:
    6. Decline to answer:
29. How many people do you feel you can communicate with regarding your feelings about performing euthanasia at work?
    1. Relatives:
    2. Friends:
    3. Work peers:
    4. Other:
    5. Not applicable:
    6. Decline to answer:
30. Do you know what mental health care resources are available in your community?
    1. Yes
    2. No
    3. Not applicable
    4. Decline to answer
31. Age in years
32. Gender
    1. Man
    2. Woman
    3. Other
    4. Decline to answer question
33. Highest level of formal education - Selected Choice
    1. No high school diploma. Please specify number of years of schooling:
    2. High school diploma
    3. Some college
    4. Bachelor’s degree
    5. Veterinary school
    6. Post graduate degree (other than veterinary school).
    7. Please specify: Decline to answer question
34. Ethnicity
    1. Hispanic or Latino
    2. Non-Hispanic or Latino
    3. Decline to answer question
35. How long have you been employed at this company?
    1. Years:
    2. Month:
    3. Weeks:
    4. Decline to answer question
36. Prior to this farm, were you employed in other sow farms on the US? If yes, total years employed in sow farms in the US: _____ years _____months - Selected Choice
    1. Yes :
    2. No
    3. Decline to answer question
37. Country of origin:
38. Native language:
39. Second language proficiency (please specify language in the textbox): - Selected Choice
    1. I speak it very well :
    2. I understand it but have trouble speaking it :
    3. I don’t understand it very well :
    4. None
    5. Decline to answer question
40. Please leave any additional comments if you have any feedback or would like to discuss anything else regarding any of the questions in this survey that may not have been addressed directly.
